# Dr. Charles P. Pengra

**Published:** 1892-03

**Authors:** 


### Obituary.

### DR. CHARLES P. PENGRA.

DR. CHARLES P. PENGRA died at his home, No. 130 Dartmouth Street, Boston, Mass., at one o'clock A.M., January 31, 1892, aged thirty-one.

The earlier portion of his life was spent in Michigan, and at the age of nineteen he had completed the course of study in the Medical Department of the University of Michigan; but on account of his age his degree was withheld until the following year, 1881.

In 1883 he received the degree of Pharmaceutical Chemist from the same university.

During 1881–84 he was assistant to Professor V. C. Vaughn, University of Michigan, the eminent physiological and sanitary chemist.

In 1884 he assumed the chair of Materia Medica and Botany in the Massachusetts College of Pharmacy, Boston.

He was for the past five years Professor of Dental Histology and Microscopy in the Boston Dental College. Both of these positions he held at the time of his death.

He was a Fellow of the American Association for the Advancement of Science, and of the Massachusetts Medical Society, and was a member of the Convention for Revision of the United States Pharmacopœia, 1890.

He was an original investigator, being especially known for his researches upon the purification of water by freezing.

The frequent contributions which have appeared in various scientific journals from his pen have made his name familiar.

Professor Pengra was a favorite with his classes, being clear, thorough, and possessed of a rare faculty of interesting his students in the subjects which he taught, and the Faculty of the Boston Dental College feel that they have lost an able instructor and efficient working member of the teaching force. Therefore, be it

*Resolved*, That this expression of their appreciation of Professor Pengra's valuable services, together with the above memorial, be spread upon the college records, and also that a copy be transmitted to his surviving wife, to whom the Faculty tender their sincere sympathy.

*Resolved*, also, That a copy be sent to the leading dental journals for publication.

George F. Eames,
S. P. S. Sharpless,
Joseph King Knight,
*Committee for the Faculty.*

